# TiO_2-x_/TiO_2_-Structure Based ‘Self-Heated’ Sensor for the Determination of Some Reducing Gases

**DOI:** 10.3390/s20010074

**Published:** 2019-12-21

**Authors:** Simonas Ramanavicius, Alla Tereshchenko, Renata Karpicz, Vilma Ratautaite, Urte Bubniene, Audrius Maneikis, Arunas Jagminas, Arunas Ramanavicius

**Affiliations:** 1 Center for Physical Sciences and Technology, Sauletekio av. 3, LT-10257 Vilnius, Lithuania; simonas.ramanavicius@gmail.com (S.R.); renata.karpicz@ftmc.lt (R.K.); vilma.ratautaite@gmail.com (V.R.); urte.bubniene@gmail.com (U.B.); andrius.maneikis@ftmc.lt (A.M.); arunas.jagminas@ftmc.lt (A.J.); 2 Department of Experimental Physics, Faculty of Mathematics, Physics and Information Technologies, Odesa National I.I. Mechnikov University, Pastera 42, 65023 Odesa, Ukraine; alla_teresc@onu.edu.ua; 3 Department of Physical Chemistry, Institute of Chemistry, Faculty of Chemistry and Geosciences, Vilnius University, Naugarduko 24, LT-03225 Vilnius, Lithuania

**Keywords:** nonstoichiometric titanium oxides, TiO_2-x_/TiO_2_, Ti_3_O_5_/TiO_2_, photoluminescence, anatase, rutile, sensor for reducing gases

## Abstract

In this research we report the gas-sensing properties of TiO_2-x_/TiO_2_-based hetero-structure, which was ‘self-heated’ by current that at constant potential passed through the structure. Amperometric measurements were applied for the evaluation of sensor response towards ethanol, methanol, n-propanol and acetone gases/vapours. The sensitivity towards these gases was based on electrical resistance changes, which were determined by amperometric measurements of current at fixed voltage applied between Pt-based contacts/electrodes deposited on the TiO_2-x_/TiO_2_-based layer. X-ray diffraction (XRD) analysis revealed the formation of TiO_2-x_/TiO_2_-based hetero-structure, which is mainly based on Ti_3_O_5_/TiO_2_ formed during the hydro-thermal oxidation-based sensing-layer preparation process. Additionally, photoluminescence and time-resolved photoluminescence decay kinetics-based signals of this sensing structure revealed the presence of TiO_2_ mainly in the anatase phase in the TiO_2-x_/TiO_2_-based hetero-structure, which was formed at 400 °C annealing temperature. The evaluation of TiO_2-x_/TiO_2_-based gas-sensing layer was performed at several different temperatures (25 °C, 72 °C, 150 °C, 180 °C) and at these temperatures different sensitivity to the aforementioned gaseous materials was determined.

1. Introduction

Among many other inorganic semiconductor-based structures, TiO_2_-based structures are often used for the development of gas-sensing devices due to their sensing properties [1]. Titanium dioxide (TiO_2_) is an n-type semiconductor, which exists in three main phases (i) anatase, (ii) rutile and (iii) brookite with bandgaps of 3.02, 3.23, and 2.96 eV, respectively [2]. All phases of TiO_2_ can be relatively easily synthesized and/or converted from each to another by relatively simple thermal treatment (annealing).

The attractiveness of TiO_2_ has significantly increased since its water-splitting ability under ultraviolet (UV) light irradiation was discovered [3]. The discovery of the latter effect facilitated the exploitation of catalytic TiO_2_ properties in the design of sensors for gases and gaseous materials. Due to remarkable properties, many different TiO_2_-based structures have found applications in various technological areas including biosensors [4,5] and chemical sensors [6,7,8,9,10].

TiO_2_-based gas sensors are not expensive. They mostly require simple analytical signal detection and evaluation systems and offer good sensitivity towards many gases and gaseous materials, including both major types of gases: (i) reducing gases such as H_2_S, H_2_, CO, NH_3_, CH_3_OH, C_2_H_5_OH, and many others volatile organic compounds (VOCs), and (ii) oxidizing gases such as O_2_, NO_2_, CO_2_ [11,12,13]. When TiO_2_-based sensors are affected by different types of gases (reducing or oxidizing) such sensors generate opposite (positive or negative) current/(electrical resistance)-based response, which depends on the type of the gas. Electrical resistance measurement-based registration is one of the simplest analytical signal-registration method in comparison to many other analytical techniques, e.g., (i) the determination based on photoluminescence (PL) measurements [14] or (ii) based on more sophisticated potentiodynamic electrical/electrochemical techniques such as impedance spectroscopy [15], etc., which can be applied for analytical signal registration in the case of TiO_2_-based sensors.

The sensing mechanism of TiO_2_-based gas sensors is complex and it can be described by the superposition of several multi-step processes: (i) a gas adsorption/desorption on TiO_2_ surface, which dependent on the nature of the gas is followed by enrichment or depletion of upper layer of TiO_2_-based structure by electrons, which significantly (ii) changes the conductivity of TiO_2_-based grains and (iii) the charge transfer between the grains. In these aforementioned processes the surface-to-volume ratio of grains, the grain size and the Debay length are playing a crucial role in the charge transfer ability of TiO_2_-based layers. Therefore, the formation of nanostructured TiO_2_ with a very high number of grains, i.e., a high number of boundaries between grains and increased surface on which gases of interest can be adsorbed, are advantageous for the development of gas sensors [12,16,17,18,19,20]. Even the sorption of gases on the surface of TiO_2_-based structures is not a very simple phenomenon, which is mostly based on several different phases, particularly physical-adsorption and chemisorption [21]. During the physical adsorption phase gas molecules (mostly oxygen), which are initially adsorbed on the surface of TiO_2_ from air, are replaced by molecules of other gases, which are present in gaseous specimen. This process is mainly determined by Van der Waals and electrostatic interactions between TiO_2_ and the adsorbed gas molecules. During the next phase, adsorbed molecules dependently on their structure and/or orientation on the surface are either attracting or donating electrons to the TiO_2_ surface layer, which induces the variation of conductivity by the aforementioned mechanism.

Despite some recent developments in TiO_2_-based hetero-structure based sensors, n-type pure TiO_2_-based gas sensors are characterized by relatively high resistance and relatively poor sensing activity, therefore, various TiO_2_-based hetero-structures are used instead of pure TiO_2_. Coupling of TiO_2_ with various materials can result in increased sensing-ability. The most promising structures are based on coupling of TiO_2_ with other semiconducting materials, which can increase sensitivity, alternate selectivity, reduce response time, lower operational temperatures in comparison to pure TiO_2_-based sensors [17,18,19,20,21,22,23,24,25].

One option of modification and/or formation of hetero-structures, which are sensitive to various gaseous materials, is related to the application of conducting polymers such as polypyrrole (Ppy), which is used in order to form TiO_2_/Ppy-based hetero-structures suitable for the determination of liquefied petroleum gases (LPG) such as propane and butane [26]. TiO_2_/Ppy-based sensors operated at relatively low temperatures, which are close to the room temperature. Some other authors have demonstrated the sensitivity of ultra-thin TiO_2_/Ppy-based hetero-structures towards NH_3_ gas [27], which was advanced towards much higher sensitivity by another research team [28]. Advancement in the application of TiO_2_/Ppy hetero-structures is attributed to the formation of n-p hetero-junctions between TiO_2_ and Ppy layers. Similar hetero-junctions were reported for TiO_2_ and another conducting polymer polyaniline (PANI) based hetero-structures TiO_2_/PANI, which were sensitive towards NH_3_ [27,29,30,31].

The primary aim of this research is to demonstrate the ability to form layer of TiO_2-x_/TiO_2_-based hetero-structures, which will be suitable for the design of gas sensor operating at relatively low temperatures. The next aim was to demonstrate that relatively low resistance of formed TiO_2-x_/TiO_2_ layer can be applied for ‘self-heating’ of the sensing structure and to evaluate how the selectivity and sensitivity of the here designed gas sensor depends on the temperature of TiO_2-x_/TiO_2_-based sensing-structure.

## 2. Experimental

### 2.1. Formation of TiO_2_ Sample

**Figure 1:**
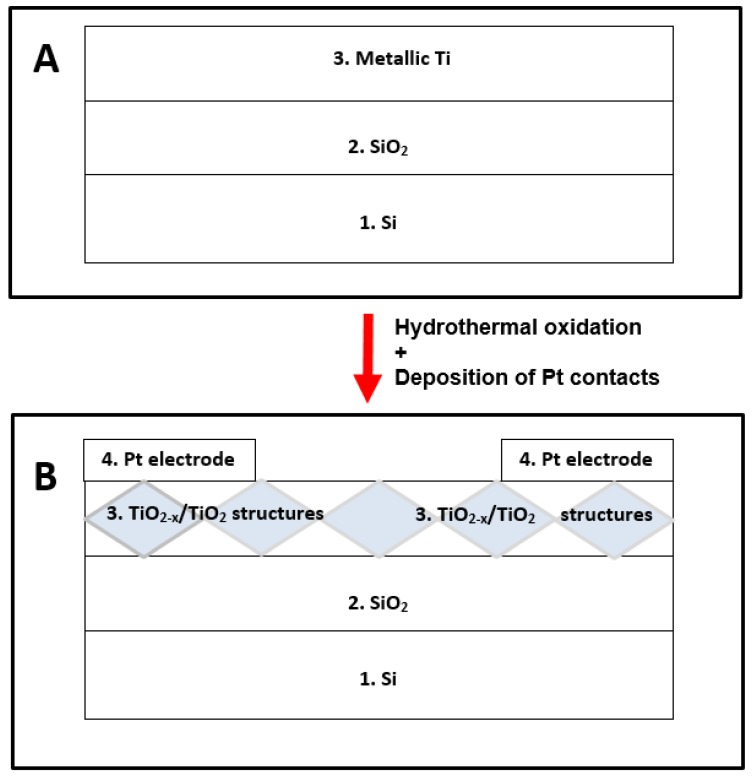
(**A**) Schematic view (layer-by-layer) of initial structure, which was used for the design of sensor: 1—Si-based wafer; 2—thin layer (100 nm) of SiO_2_; 3—thin layer (100 nm) of Ti deposited by magnetron sputtering. (**B**) Schematic view (layer-by-layer) of TiO_2-x_/TiO_2_-based sensor structure: 1—Si-based wafer; 2—thin layer (100 nm) of SiO_2_; 3—nonstoichiometric TiO_2-x_/TiO_2_ layer formed by hydrothermal oxidation; 4—Pt electrodes/(contact zones) deposited by magnetron sputtering.

A Si wafer (1) was oxidized in electric oven with increased concentration of oxygen to form a few micrometers thick oxide (SiO_2_) layer (2) over the Si wafer. After that the metallic titanium (Ti) layer (3) of 100 nm thickness was sputtered by a magnetron. Amorphous non-stoichiometric titanium oxide (TiO_2-x_) and titanium dioxide (TiO_2_) based hetero-structure (TiO_2-x_/TiO_2_) was formed by hydrothermal oxidation of Si/SiO_2_/Ti-based wafer in aqueous alkaline solution. Finally, platinum (Pt) electrodes/(contact zones) (Figure 1, Section 4) were formed on the top of the crystalline TiO_2_ by magnetron sputtering.

The next step in sensor development procedure was based on annealing of Ti-based structures, which was performed at three different temperature regimes:

(i) 50 °C/1 h + 400 °C/2 h temperature for the formation of TiO_2-x_/TiO_2_ (400 °C) structure;
(ii) 50 °C/1 h + 600 °C/2 h temperature for the formation of TiO_2-x_/TiO_2_ (600 °C) structure;
(iii) 50 °C/1 h + 800 °C/2 h temperature for the formation of TiO_2-x_/TiO_2_ (800 °C) structure.

All the aforementioned procedures enabled us to form mixed crystal phase TiO_2-x_/TiO_2_-based hetero-structures (Figure 1, layers 4 and 5) at over-layer containing significant amount of TiO_2_ in the form of anatase and/or rutile phases and TiO_2-x_, which was formed in the deeper layers of the structure below fully oxidized stoichiometric TiO_2_.

Finally, platinum-based electrodes were deposited on the formed TiO_2-x_/TiO_2_ (400 °C), TiO_2- x_/TiO_2_ (600 °C) and TiO_2-x_/TiO_2_ (800 °C) layers by magnetron sputtering. The dimensions of the structure were: 8 mm—length of the structure and 3 mm—distance between platinum electrodes deposited over the TiO_2-x_/TiO_2_-based layer. The thickness of formed titanium layer was 100 nm, the measurements were performed at 25 °C (room temperature), 72 °C, 150 °C, 180 °C. The humidity of the supplied aforementioned gaseous materials containing air stream was constant during all parts of measurements with methanol, ethanol, n-propanol, and acetone vapour: partial pressure of water in the air stream was 3.170 kPa, which corresponds to 100% of relative humidity at 25 °C and at 101.325 kPa pressure. During the measurement of signals towards water, initially a dry air stream was supplied and mixed with 1170 ppm of H_2_O containing air stream at corresponding ratio.

Platinum (Pt) contacts were formed by magnetron sputtering using sputter from VSTSER (Tel-Aviv, Israel) in direct current (DC) regime under Argon (Ar) atmosphere (20 mTorr pressure) using 1” diameter and 99.99% purity Pt target. Contacts geometry was determined by a mask. In order to improve adhesion between TiO_2-x_/TiO_2_-based layer and platinum at first a thin (20 nm) titanium (Ti) layer was sputtered on which Pt contacts were formed. Sputtering power for Ti layer formation was 2.55 W/cm^2^ with the growth of 0.13 nm/s. During the formation of the Pt procedure, magnetron power was 3.06 W/cm^2^, with layer growth of 0.08 nm/s.

### 2.2. Scanning Electron Microscopy (SEM)-Based Characterization of Formed TiO_2-x_/TiO_2_-Based Hetero-Structure

The structural properties of the obtained TiO_2_ samples on silicon substrates were determined using a scanning electron microscope (SEM) Helios NanoLab 650 from FEI (Eindhoven, The Netherlands).

### 2.3. X-ray Diffraction (XRD) Characterization of TiO_2-x_/TiO_2_-Structure

The phase composition of thin films was determined by X-ray diffractometer D8 Advanced from Bruker (USA) with grazing-incident X-ray diffraction (XRD) over a 2θ range of 22°–65° using Cu Kα (= 1.5046). Films for XRD investigations were based on: (i) metallic Ti (100 nm) layer deposited over Si substrate covered by 300 nm SiO_2_ layer (ii) TiO_2−x_/TiO_2_ (400 °C) hetero-structure, which was formed by the oxidation of the same metallic Ti layer (mentioned in part ‘i’) according to the aforementioned protocol of hydrothermal oxidation at 400 °C.

The diffraction pattern was generated by X-ray beam at grazing incidence angle of 2°. XRD data library ‘CDD Database PDF2010-PDF-2/Release 2010 RDB’ was applied for the analysis of XRD patterns.

TiO_2_-powder, which by producer was declared as TiO_2_ anatase (TiO_2(anatase)_) phase, was received from Sigma-Aldrich (St. Louis, United States) and was used as XRD control while was investigated at the same grazing incidence angle of 2° over similar 2θ range of 22°–65°.

### 2.4. Photoluminescence (PL)-Based Characterization of TiO_2-x_/TiO_2_-Structure

Optical properties of TiO_2-x_/TiO_2_-based hetero-structures deposited on oxidized silicon substrates were investigated by photoluminescence (PL) studies using Edinburgh-F900 spectrophotometer (Edinburg Instruments Ltd., Livingston, UK). The photoluminescence spectra of TiO_2-x_/TiO_2_-based hetero-structures were excited by solid-state laser with an excitation wavelength of 375 nm (the average pulse power was about 0.15 mW/mm^2^, the pulse duration 70 ps) and photoluminescence was measured in the range of 400–700 nm. For comparison, the photoluminescence spectra of similar oxidized silicon substrates were also registered. All fluorescence spectra were corrected accounting the sensitivity of the instrument.

Photoluminescence measurements of ‘self-heated’ structure were performed at different voltages that heated the sensor up to particular temperatures. The exact temperature was followed with a thermocouple attached to the TiO_2-x_/TiO_2_ hetero-structure.

The position and intensity of the photoluminescence maximum was determined as corresponding characteristics of a Gauss function using the Origin program.

### 2.5. Determination of Analytical Signal towards Reducing Gases by TiO_2-x_/TiO_2_-Structure Based Sensor

Measurements of current passing through the sensor structure were performed by potentiostat/galvanostat Autolab 30 Eco Chemie Gmbh (Utrecht, The Netherlands), which was controlled by NOVA software. The TiO_2−x_/TiO_2_ (400 °C) sample was investigated under different constant voltages, which were applied by potentiostat on platinum-based electrodes deposited on the TiO_2-x_/TiO_2_ (400 °C) layer.

Evaluated gas concentrations were fixed at: 105 ppm for water, 118 ppm for methanol, 53 ppm for ethanol, 18 ppm for n-propanol, 220 ppm for acetone.

**Figure 2:**
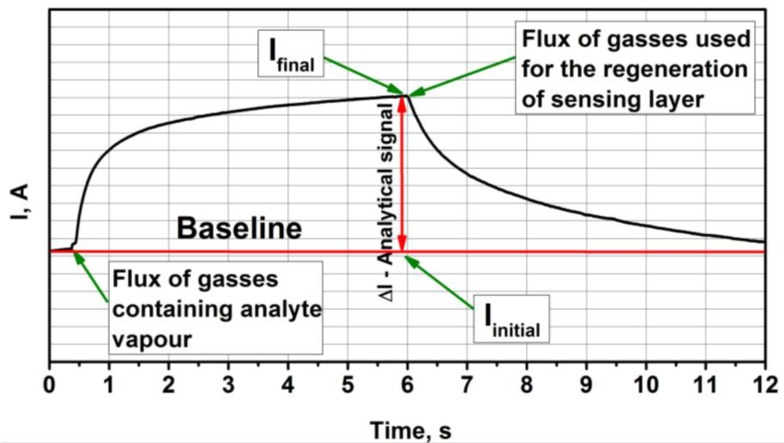
Representation of typical analytical signal registered by TiO_2-x_/TiO_2_ (400 °C)-based structure. It should be noted that Δ*I,* the duration of signal development and the regeneration of sensor after measurements were different for different gases and different concentrations of those gases.

Electrical resistance of TiO_2−x_/TiO_2_ (400 °C)-based gas-sensitive structure decreased due to their increased conductivity, therefore, current passing through TiO_2-x_/TiO_2_ (400 °C)-based structure increased. In present research we have registered a variation of current (Δ*I*) at constant potential. These variations of measured current (*I*) (Figure 2) were converted into resistance (*R*) of TiO_2-x_/TiO_2_-based structure according to Ohms law:

*R* = *V*/*I* (1)

where *V* is applied voltage.

Then relative response (Δ*R*, %) was calculated as:

Δ*R* = 100 × (*R*_final_ − *R*_initial_)/*R*_initial_ (%) (2)

where *R*_initial_ is initial resistance of TiO_2-x_/TiO_2_ (400 °C)-based structure calculated from *I*_initial_ at baseline, *R*_final_—final resistance calculated from *I*_final_ (Figure 2).

### 2.6. Determination of Electrical Resistance Variation with Temperature

Measurements of resistance vs. temperature, were performed by system based on closed cycle helium cryostat made by Sumitomo Heavy Industries (Tokyo, Japan) combined with RDK-408D 4K cold head and SRDK Series cryocooler and CSA-71A compressor unit. Temperature was controlled by Lakeshore 340 temperature controller (Lake Shore Cryotronics, Inc., Westerville, OH, USA). Resistance was measured by Tektronix DMM 4050 multimeter (Tektronix UK Ltd., Bracknell, UK). This closed-cycle helium cryostat was used for measurement of resistance and was performed only after exact equilibration of temperature at each selected point in the temperature range from 4.2 K to 310 K, with 5 K intervals between measurement points. Temperature was changed in cyclic manner in two ways: black cycles shows points measured by cooling down, red squares shows points by increasing temperature. Measured performed in vacuum 10^−3^ Torr.

## 3. Results and Discussion

### 3.1. SEM-Based Structural Characterization of TiO_2-x_/TiO_2_-Based Layer

**Figure 3:**
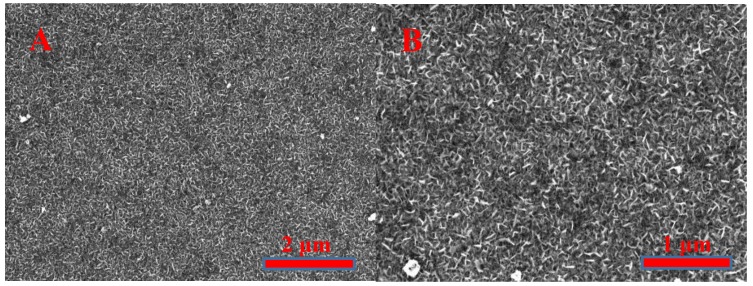
Scanning electron microscope (SEM) images of TiO_2-x_/TiO_2_ (400 °C)-based hetero-structure at different magnification: (**A**)—at ×25000; (**B**)—at ×50000.

SEM images of TiO_2-x_/TiO_2_ (400 °C)-based hetero-structure at different at some extent s (Figure 3A,B) illustrate that the sample has a highly porous surface with nanostructures in the form of nano-plates and nano-sponges, which significantly enhanced both (i) surface area and (ii) surface to volume ratio of the gas-sensitive area of sensor. Therefore, such formations are very beneficial in order to obtain increased surface area, which is available for gas adsorption and enhances the sensitivity of such a structure.

### 3.2. XRD Characterization of TiO_2-x_/TiO_2_-Structure

**Figure 4:**
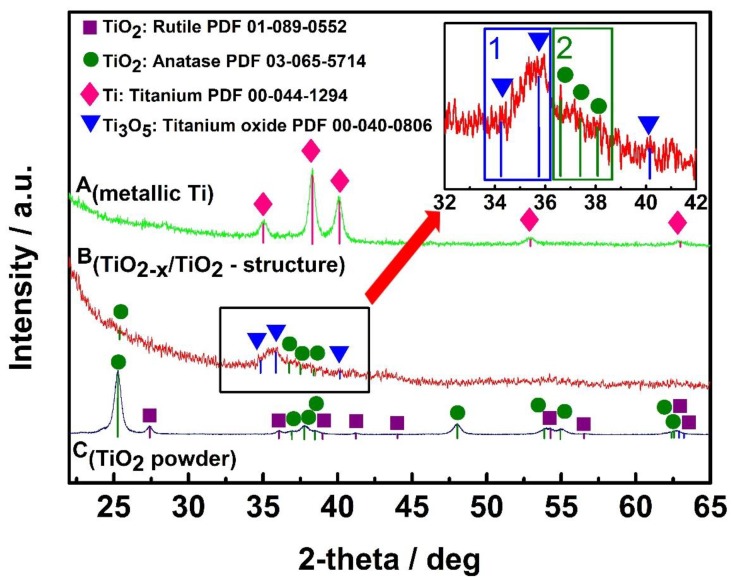
X-ray diffraction (XRD) patterns of: A_(metallic Ti)_—metallic Ti layer of 100 nm thickness, which was formed by magnetron sputtering; B_(TiO2-x/TiO2-structure)_— TiO_2-x_/TiO_2_ (400 °C)-based hetero-structure, which was formed from above mentioned metallic 100 nm thick Ti layer; C_(TiO2 powder)_—TiO_2_-powder, which was used as ‘control sample’ and by supplier (Sigma-Aldrich) was declared as 99.3% TiO_2_ in the anatase phase.

The XRD pattern of metallic Ti (Figure 4, A_(metallic Ti)_), which was observed for initial metallic Ti layer of 100 nm thickness that was formed by magnetron sputtering and was used for further hydrothermal oxidation at 400 °C into a TiO_2-x_/TiO_2_ (400 °C)-based hetero-structure, represents all characteristic peaks of metallic Ti well (according to PDF ‘00-044-1294 for metallic Ti’).

The XRD pattern of TiO_2-x_/TiO_2_ (400 °C) (Figure 4, B_(TiO2−x/TiO2-structure)_) represents relatively high dispersion in TiO_2-x_/TiO_2_ (400 °C)-based hetero-structure, but according to Fukushima et al. [32] even the presence of broad ‘peak area’ in titanium oxide based pattern between 27° and 37° can be assigned to the presence of Ti_3_O_5_, Ti_4_O_7_ and/or Ti_8_O_15_. Thus, in our research we have observed a much better expressed XRD peak between 34° and 37° (Figure 4, Inset, area 1), which is in good agreement with γ-Ti_3_O_5_ reported in the PDF ‘00-040-0806 for γ-Ti_3_O_5_’ and also in relatively good agreement with the XRD pattern presented by Yoshimatsu et al. [33] for γ-Ti_3_O_5_ formed by pulsed laser deposition. Results obtained by Yoshimatsu et al. [33] showed low-temperature superconductivity in both Ti_4_O_7_ and γ-Ti_3_O_5_ films. In addition, these authors reported relatively broad XRD peaks at 36–38° for γ-Ti_3_O_5_ and at 42–43° for Ti_4_O_7_. The peak observed in our research (Figure 4, B_(TiO2−x/TiO2-structure)_ and Inset, area -1) according to the shape-like-features and signal to noise ratio is very similar to that at 36–38° for γ-Ti_3_O_5_, but just some shift is observed because preparation procedures of both films were very different, therefore, the composition and stoichiometry of both TiO_2-x_-based structures discussed here can be different at some extent. Another broad peak area (Figure 4, Inset, area 2) between 36° and 39° is assigned to TiO_2(anatase)_ according to PDF ‘01-075-2545 for TiO_2(anatase)_’ and control XRD pattern (Figure 4, C_(TiO2 powder)_) registered at the same experimental conditions for TiO_2(anatase)_ powder purchased from Sigma Aldrich, where according to match between XRD patterns (Figure 4. C_(TiO2 powder)_) with PDF ‘01-089-0552 for TiO_2(rutile)_’ we have detected the presence of some TiO_2(rutile)_ phase.

**Figure 5:**
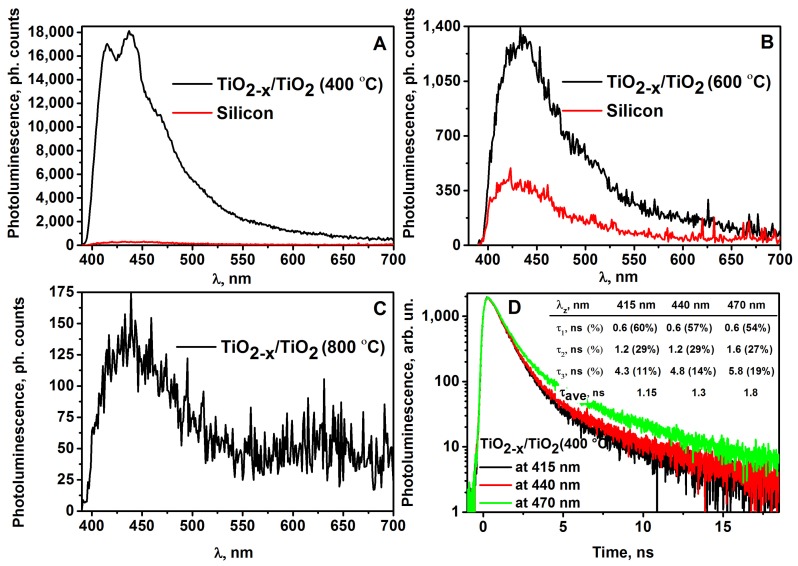
(**A**) Photoluminescence spectra of TiO_2-x_/TiO_2_ (400 °C) sample (1), and silicon substrate (2); (**B**) Photoluminescence spectra of TiO_2-x_/TiO_2_ (600 °C) sample (1), and silicon substrate (2); (**C**) Photoluminescence spectra of TiO_2-x_/TiO_2_ (800 °C) sample; (**D**) Photoluminescence decays of TiO_2-x_/TiO_2_ (400 °C) sample at different photoluminescence emissions bands under 375 nm excitation.

Titanium pentoxide (Ti_3_O_5_) with polymorphisms (α-, β-, γ-, δ-, and λ-phases) is ‘a close neighbour’ of the Magnéli phase [34,35,36,37,38], and sometimes it is designated as the first member of the Magnéli phase, because their chemical formula is consistent with that of the Magnéli phase (TinO_2n-1_ at n = 3). According to PDF ‘00-040-0806 for γ-Ti_3_O_5_′, Ti_3_O_5_ possesses a monoclinic cell (a = 9.9701 Å, b = 5.0747 Å, c = 7.1810 Å, β = 109.865°), which is superconducting at low temperatures (below 3 K) similarly to Ti_4_O_7_-based Magnéli phase [33]. Differently from the ‘most respectful’ member of Magneli phase—Ti_4_O_7_, which in crystal structure is having TiO_2(rutile)_-based shear planes [39,40], in Ti_3_O_5_ there are no such TiO_2_-rutile based shear planes [33]. Therefore, in our XRD patterns we are observing only signs of M_3_O_5_ and TiO_2(anatase)_ without any presence of rutile. Such a composition was formed because for the formation of TiO_2-x_/TiO_2_-based hetero-structures we have applied 400 °C temperature at which the formation of M_3_O_5_-TiO_2(anatase)_ ‘intergrowths’ is observed, as it has been reported and investigated by other research teams [41] and very recently has been confirmed by some other research group in different conditions [42]. The formation of above mentioned M_3_O_5_-TiO_2(anatase)_ ‘intergrowths’ is in well agreement with our results based on photoluminescence (Figure 5A) and photoluminescence decay (Figure 5D) measurements, which are discussed in the next chapter, where we are clearly observing effects induced by the presence of TiO_2(anatase)_.

### 3.3. Photoluminescence Properties of Hybrid TiO_2-x_/TiO_2_-Based Structures

All photoluminescence spectra of the evaluated TiO_2-x_/TiO_2_-based structures were characterized by a wide photoluminescence maximum in the region of wavelengths between 415–500 nm. However, the quality of the photoluminescence signals of all three TiO_2-x_/TiO_2_-based structures were very different. As it is demonstrated in the Figure 5, this depends on the temperature that is applied for the annealing of TiO_2-x_/TiO_2_-based structures, because at different temperatures different phases of TiO_2_ on the surface of TiO_2-x_/TiO_2_-based structures were formed. TiO_2-x_/TiO_2_(400 °C) structure, which was formed by annealing at 400 °C, generated the most intense photoluminescence signal (Figure 5A). The upper layer generates the strongest photoluminescence signal and the spectrum of TiO_2-x_/TiO_2_ (400 °C) is characterized by photoluminescence peaks, which are observed at 415, 440, 470 nm of the main photoluminescence band (Figure 5A). These photoluminescence peaks reveal the presence of TiO_2(anatase)_ in TiO_2-x_/TiO_2_ (400 °C) hybrid-structure. TiO_2-x_/TiO_2_ (600 °C) structure, which was formed by annealing at 600 °C, also has demonstrated some photoluminescent properties but the photoluminescence signal was about 10 times lower (Figure 5B) and revealed presence of mixed TiO_2(anatase)_ and TiO_2(rutile)_ structures of TiO_2-x_/TiO_2_ (600 °C) sample.

TiO_2-x_/TiO_2_ (800 °C) structure, which was formed by annealing at 800 °C, was characterized by very weak, about 100 times lower photoluminescence signal, than that was observed for TiO_2-x_/TiO_2_ (400 °C) sample (Figure 5C). Thus TiO_2-x_TiO_2_ (800 °C) structure is not suitable for further investigations required for optoelectronic sensors and, as revealed by the results below, this structure was also not suitable for the sensing of gases selected for this research.

**Figure 6:**
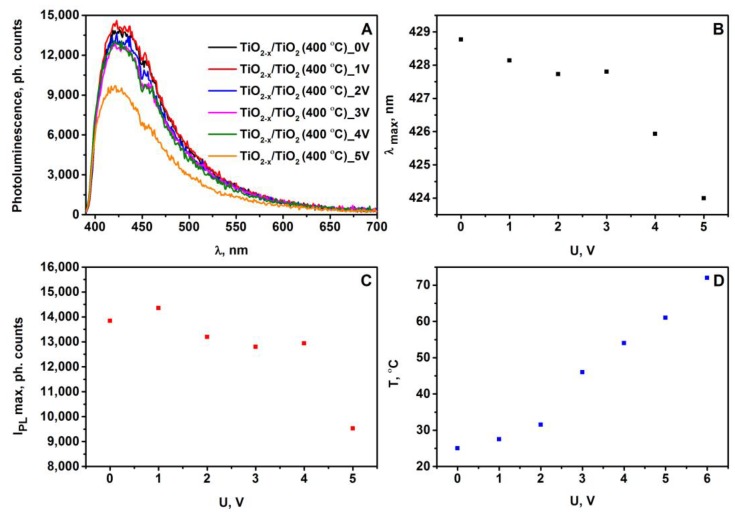
(**A**) Photoluminescence spectra of TiO_2-x_/TiO_2_ (400 °C)-based sample at different applied voltage; (**B**) The changes in spectral position of photoluminescence maximum vs. voltage of TiO_2-x_/TiO_2_ (400 °C)-based sample; (**C**) The changes in photoluminescence maximum intensity vs. voltage of TiO_2-x_/TiO_2_ (400 °C)-based sample; (**D**) The plot of temperature vs. voltage of TiO_2-x_/TiO_2_ (400 °C)-based sample.

TiO_2-x_/TiO_2_ (400 °C) structure showed the most interesting and the highest quality photoluminescence signal. Therefore, during the next experiment, which was also based on photoluminescence spectrum registration, this structure was investigated under different constant voltages, which were applied by potentiostat on platinum-based electrodes deposited on the TiO_2- x_/TiO_2_ (400 °C)-based layer. The plots of photoluminescence spectra (Figure 6A) vs. applied voltage are shown in Figure 6B. The elevated voltage from 1 V to 5 V, results the decrease of intensity of the main photoluminescence maximum and a small shift of photoluminescence maximum position towards shorter wavelengths.

Figure 6B,C show the position and the intensity of the photoluminescence maximum, which is changing depending on the voltage applied to TiO_2-x_/TiO_2_ (400 °C)-based sample. The position (Figure 6B) of the photoluminescence maximum was determined as a maximum of Gauss function, which represented the best fitting with registered photoluminescence spectra. At voltage values (from 1 V to 5 V) tested in this experiment the spectral position of the photoluminescence maximum (λ_max_) gradually shifts towards the infrared (IR) region (Figure 6B). In general, the difference between the positions λ_max_ for TiO_2-x_/TiO_2_ (400 °C)-based sample at 0 V and 5 V is about 5 nm. A similar trend related to the decrease in photoluminescence intensity of the main maximum with increasing voltage was observed (Figure 6C). The decrease of photoluminescence intensity by the increase of applied voltage from 0 V to 5 V has been determined, and it shows that the concentration of photoluminescence emitting centers in TiO_2-x_/TiO_2_ (400 °C) hetero-structure is reducing by increasing voltage. The plot of temperature vs. voltage of TiO_2-x_/TiO_2_ (400 °C)-based sample (Figure 6D) illustrates dependence in tested potential interval, which can be predicted from Ohms law, followed by basic recalculations into the heat released by this system.

Such behavior of the photoluminescence spectra vs. applied voltage can be caused by an increased sample temperature, which is dependent on electrical current flowing through the TiO_2- x_/TiO_2_ (400 °C)-based hetero-structure. Thus, a rapid decrease in the photoluminescence intensity of TiO_2_ with an increase in voltage at U ≥6 V is related to the increase of the sample temperature, which leads to the temperature-based quenching of photoluminescence of TiO_2(anatase)_ [43,44,45]. Photoluminescence studies of nanostructured TiO_2_ at room-temperature were reported in some other research: (i) the photoluminescence of TiO_2(anatase)_ colloidal particles of different sizes occurs from the shallow trap levels, which are located between 0.41 and 0.64 eV below the conduction band [46]; (ii) the narrow photoluminescence emission bands of TiO_2(anatase)_ powder, which originated from the self-trapped exciton emission (STE) in crystalline TiO_2(anatase)_ containing TiO_6_ octahedral sheet-based structures, was reported [47,48]. In addition to registration of photoluminescence spectra, we have measured the photoluminescence decay kinetics for TiO_2-x_/TiO_2_ (400 °C)-based hetero-structure at different narrow photoluminescence emission bands (Figure 5D). The photoluminescence of TiO_2(anatase)_ decay non-exponentially with dominating fast component, which is characterized by a decay of about 0.6 ns. The photoluminescence decay kinetics presented here clearly verify the self-trapped exciton emission origin of photoluminescence, which from the broad range of non-stoichiometric titanium oxides (TiO_2−x_) and stoichiometric titanium oxides (TiO_2_) is the most characteristic for crystalline TiO_2(anatase)_ [49].

### 3.4. Electrical Resistance Variation with Temperature

**Figure 7:**
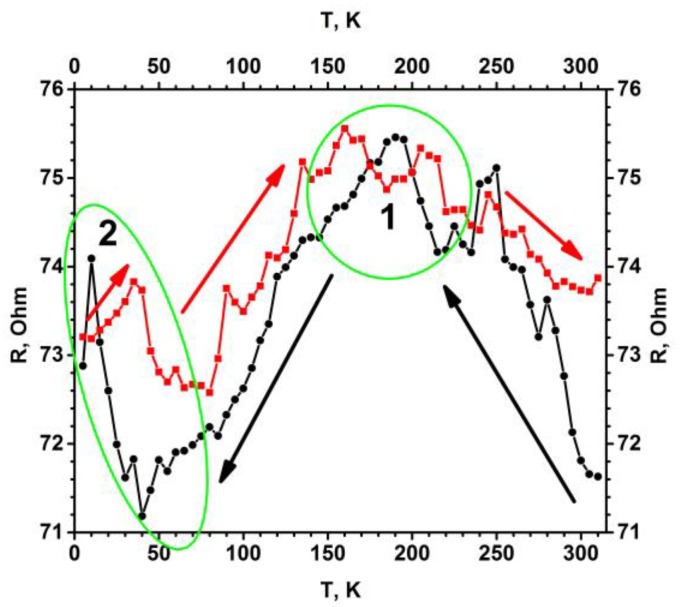
Temperature dependence of electrical resistance (R(T)) for the TiO_2-x_/TiO_2_ (400 °C)-based hetero-structure. Temperature was changed in two ways (indicated by black and red arrows): (i) black cycles shows points measured by cooling down, (ii) red squares shows points by increasing temperature. Measured was performed in vacuum using helium cryostat.

Electrical resistance measurements of TiO_2-x_/TiO_2_-based structure revealed the oxidation of metallic titanium-based layer, because by thermal oxidation the resistance of the sample has increased from 0 Ω (for bare Ti-based layer) up to 72 Ω (for formed TiO_2-x_/TiO_2_-based structure). The increase of resistance together with the shift of photoluminescence signals clearly shows the formation of TiO_2_ in the phase of anatase and rutile. However, stoichiometric TiO_2_-based layers have relatively low electrical conductivity, which is typically of 10^−10^ S/m, but it was demonstrated that the conductivity of TiO_2_-based layers can be significantly increased by heat-based treatment at a high temperature in a reducing gas-based environment [50]. Further investigations revealed the formation of TiO_2-x_/TiO_2_-based hetero-structures, because only TiO_2_ based structures have relatively high band-gap and, therefore, they do not conduct well while the presence of TiO_2-x_ in forms of Ti_2_O_3_, Ti_3_O_5_ and/or Ti_4_O_7_ significantly increases the conductivity of such hetero-structures. It should be noted, that an important issue related to TiO_2-x_ conductivity is that these oxides at the stoichiometry of Ti_n_O_2n- 1_ (with 3 < n < 10) are forming so called Magnéli phases [51], which possess some properties of metallic conductor [52,53]. As seen from resistance vs. temperature dependence (Figure 7, area 1), in the TiO_2-x_/TiO_2_-based hetero-structures at temperatures below 150–180 K reported here, we have also determined some signs of such transition into metallic conductivity, which is characteristic of presence of Magnéli phases. 

The R(T) dependence (temperature dependence of electrical resistance, Figure 7) is roughly temperature independent from 310 K down to relatively low temperatures 180–150. Such an R(T) dependence (almost constant R(T) with respect to T with some increase of conductivity when temperature is decreasing) is expected for a heterogeneous mixture of strongly disordered metal/(metal oxide)-based layer, and proves that the TiO_2-x_/TiO_2_ (400 °C)-based hetero-structure is strongly disordered and composed of many randomly oriented nanocrystals and of many different TiO_2-x_ phases and probably even some metal clusters randomly distributed in the volume of TiO_2-x_/TiO_2_-based hetero-structures. It is also remarkable that the roughly constant R(T) dependence also exhibits a series of small resistance jumps in the range of a few percent. These resistance jumps might be induced by the metal-semiconductor transitions within the involved Magneli phases, which is actually a combination of a variety of different Ti_n_O_2n-1_ phases rather than a single phase. Interestingly at 40 K the increase of electrical resistance is observed (Figure 7, area 2), similar increase, which was followed by the drop of conductivity below 4 K, observed by some other authors [33], which have reported superconductivity in Ti_4_O_7_ and γ-Ti_3_O_5_ films. Some earlier research illustrated that so called Magnéli phases can be observed in TiO_2-x_-based layers [54] as planes based on Ti_n_O_2n-1_ that are penetrating through a matrix of TiO_2_, and therefore, this shear plane based on Ti_n_O_2n-1_ structure can conduct relatively well [52,53]. There are some indications that the conductivity of properly doped or reduced TiO_2_, which partly turns into Ti_n_O_2n-1_, at low temperatures is based on n-type conductivity along the aforementioned shear planes. Similar conductivity features are exploited in memristor-type devices based on TiO_2_, in which electrical resistance is changed by the oxidation/reduction of the TiO_2_-based layer by applied corresponding potentials [55]. In other research, it has been reported that the Ti^3+^-containing TiO_2-x_/TiO_2_, has localized oxygen vacancies, which are beneficial for the electron mobility in n-type semiconducting TiO_2-x_/TiO_2_ structure [56]. We predict that the aforementioned oxygen vacancies are offering advanced gas-sensing ability for TiO_2-x_/TiO_2_ hetero-structure evaluated in our research. The reduction of TiO_2_-based layers leads to the formation of non-stoichiometric titanium oxides, which are represented by the general formula Ti_n_O_2n-1_ (where n is in the range between 3 and 10 (3 < n < 10) and are known as Magneli phases [51]. In non-stoichiometric titanium dioxide (TiO_2-x_) with a low x (0 < x < 0.10), the dominating point defects in the structure are based mainly on Ti^3+^ and Ti^4+^ interstitials and on oxygen vacancies [57]. However, the Magneli phases in which x is in the range between 0.10 and 0.34 (0.10 < x < 0.34) extended planar defects and crystallographic shear planes, which are varying according to the oxygen deficiency are observed [52,58]. TiO_2-x_/TiO_2_-based hetero-structure, which due to the formation of Ti^3+^ has Ti_n_O_2n-1_ doped TiO_2-x_ clusters (Figure 1B, 3rd layer) with significantly advanced electrical conductivity, can be synthesized by several different methods: plasma treatment [59], metallic zinc-based reduction [60], high-energy particle bombardment [61], laser irradiation [62] and some reactions at higher temperatures [63]. In addition to these methods, in recent research we have demonstrated that the hydro-thermal approach applied here is also suitable for the formation of TiO_2-x_/TiO_2_-based hetero-structures from initially deposited titanium-based layer. Due to good electrical conductivity and chemical stability, the aforementioned Magneli phases are applied in variety of applications, e.g., cathodic protection, batteries, catalyst support for fuel cells, waste and contaminated water treatment [50,64,65]. However, the majority of Magneli phases based research has been related to the fabrication of powders [19,20], only few attempts to form Magneli phase based fibers of ~250 μm with tenable conductivity have been reported [54]. By contrast with those research projects, we have produced thin layer of TiO_2-x_-based Magneli phases by oxidation of metallic titanium-based layer, which was deposited by magnetron sputtering. However, due to polymorphism of Ti_3_O_5_ based difficulties the growth of a single Ti_3_O_5_ crystal is still very challenging, therefore physical properties of Ti_3_O_5_ are still under debate [33]. Only several studies have dealt with the structural phase transitions accompanying metal–insulator transition of Ti_3_O_5_, which were induced: (i) by irradiation with visible-light pulses for β ↔ λ transition [37], (ii) at 450 K temperatures for α ↔ β transition [34] and at 240 K temperatures for δ ↔ γ transition [35,36,37,38]. In addition, metal–insulator transition around 350 K was reported by Yoshimatsu et al. [33]. Such a temperature region (240–350–450 K), where the most significant variation of Ti_3_O_5_ conductivity was observed, is in good agreement with our recent research, because we clearly observed the variation of conductivity based on the presence/absence of gaseous compounds in temperature region between room temperature (298 K) and 180 °C (453 K).

### 3.5. Gas Sensing by TiO_2-x_/TiO_2_ (400 °C) Hetero-Structure-Based Sensor

**Figure 8:**
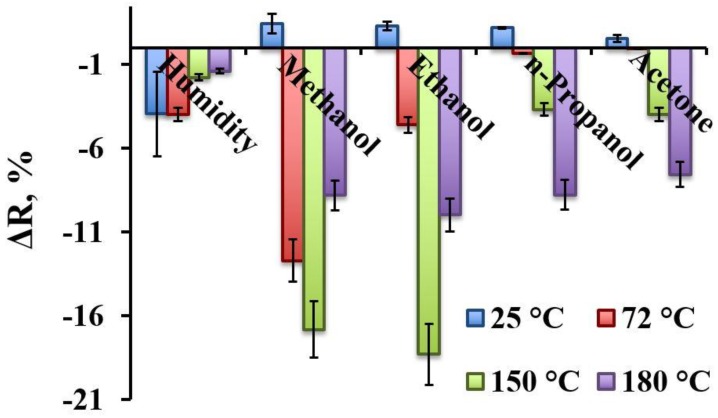
The response of TiO_2-x_/TiO_2_ (400 °C)-based hetero-structure towards humidity (H_2_O), methanol, ethanol, n-propanol, and acetone at different temperatures (25–180 °C).

Changes of electrical current passing through TiO_2-x_/TiO_2_-based hetero-structure at fixed potential were evaluated and recalculated into changes of resistance (Δ*R* %) using Equations (1) and (2). Δ*R* was evaluated as analytical signal of this sensor. Results presented in Figure 8 illustrate that already at room temperature (25 °C) the sensor shows some sensitivity towards humidity and to all four gaseous materials (methanol, ethanol, n-propanol, acetone) evaluated here. But the signals determined amperometrically at constant 0.5 V potential, which was required for the achievement of 25 °C, temperature towards humidity and towards all the aforementioned gases were very different. In the presence of water, the resistance decreased, while in the presence of methanol, ethanol, n-propanol and acetone the resistance of the sensor increased. It is observed due to different nature of these compounds. On the surface of TiO_2-x_/TiO_2_-based structure adsorbed water tends to fill the boundaries between TiO_2-x_ and TiO_2_ grains and therefore it enhances the conductivity of TiO_2-x_/TiO_2_-based hetero-structure, while all other materials have much lower conductivity in comparison to species, which they are replacing during physical- and/or chemical-sorption of methanol, ethanol, n-propanol, acetone. Therefore, the conductivity of the TiO_2-x_/TiO_2_-based structure at some extent decreases (in the range of 1% in comparison to initial conductivity of TiO_2-x_/TiO_2_-based structure). There are clear indications that sensitivity towards humidity tends to decrease by elevation of temperature from 72 °C to 180 °C, because at higher temperatures H_2_O evaporates from the surface of TiO_2-x_/TiO_2_-based hetero-structure. A similar tendency at 180 °C was observed for methanol and ethanol, but for n-propanol and acetone the highest sensitivity was observed at the highest evaluated temperature of 180 °C, while the best sensitivity towards methanol and ethanol was determined at 150 °C and the best selectivity towards methanol was achieved at 72 °C. 

Different sensitivities towards various gaseous materials at different temperatures opens the avenue to apply the array based on similar TiO_2-x_/TiO_2_-based hetero-structures, where between Pt electrodes different constant potential will be applied and this will heat these structures up to different temperatures, where TiO_2-x_/TiO_2_-based structures will have different sensitivities. Therefore, the read-out signals from such arrays can be evaluated by analysis of variance (ANOVA)-based approaches and interpreted as analytical signals. With applied ‘self-heating’ of the sensor, the best selectivity towards methanol was achieved at 72 °C, and for ethanol the temperature was 150–180 °C. For n-propanol and acetone signals increase with heating and reaches its maximum at 180 °C. Thus TiO_2_ thin films are the best for methanol detection at 72 °C, for ethanol at 150 °C and for acetone at 180 °C as the response to other gases are significantly lower. There are some indications that to all other gaseous materials investigated here, TiO_2-x_/TiO_2_-based hetero-structures were the most sensitive at even higher temperatures, but these temperatures were not available due to limitations of our experimental set up.

**Figure 9:**
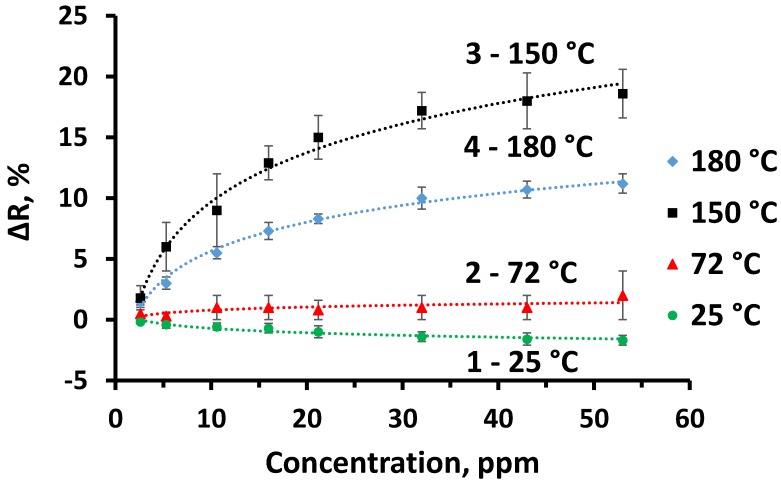
The response of TiO_2-x_/TiO_2_ (400 °C) hetero-structure towards ethanol, at different temperatures: 25 °C, 72 °C, 150 °C and 180 °C.

Figure 9 represents normalized response (Δ*R*, %) of TiO_2-x_/TiO_2_(400 °C) hetero-structure towards ethanol at four temperatures evaluated here: 25 °C, 72 °C, 150 °C and 180 °C. At 150 °C and 180 °C temperatures the limit of detection (LOD) was 2.6 ppm (Figure 9, lines 3 and 4), while at 72 °C it was 5.3 ppm and at room temperature (25 °C) it was 10.6 ppm, due to relatively low response in comparison to background noise. The coefficient of variation calculated from 12 measurements at 150 °C towards 53 ppm of ethanol, was 8.8%. During the measurements of different ethanol vapour concentrations, steady-state current was achieved within 4–20 s, dependently on ethanol concentration (4 s for the lowest measured ethanol concentration and 20 s for the highest measured ethanol concertation). It should be noted that for some volatile organic compounds evaluated here the response of sensing layer was even slower (e.g., towards the highest n-propanol concentration it exceeded 35 s). This fact can be related to: (i) some inertia of gas supply system and (ii) the dimensions of analyte-molecule larger molecules needs some more time to access deeper layers of TiO_2-x_/TiO_2_ (400 °C)-based hetero-structure.

## 4. Conclusions and Future Developments

In this research we have succeeded in forming TiO_2-x_/TiO_2_-based hetero-structure from a thin metallic titanium-based layer. This structure as a sensing layer was integrated into a gas sensor suitable for the determination of some reducing gases. Relatively high conductivity of the TiO_2-x_/TiO_2_-based hetero-structure was exploited for ‘self-heating’ of this sensor. Such ‘self-heating’ is very beneficial for the development of TiO_2-x_/TiO_2_-based sensors, because only at higher temperatures (from 72 °C to 180 °C) was advanced sensitivity of TiO_2-x_/TiO_2_-based sensor towards methanol, ethanol, n-propanol and acetone achieved. Analytical performance of the sensor proposed here can be adjusted by the optimization of TiO_2-x_/TiO_2_-based sensing structure and analytical signal registration approach, e.g., different methods of analytical signal registration (e.g., various potentiostatic, potentiodynamic and galvanostatic methods), which at the same time will serve as heating protocols, can be also applied in order to change the sensitivity/selectivity of here proposed TiO_2-x_/TiO_2_-based hetero-structure. Therefore, after some further investigations it can be applied to the design of sensors with different selectivity and sensitivity.

Despite some progress in the preparation of TiO_2_-based materials, practical exploitation of TiO_2_-based structures self-doped by Ti_n_O_2n-1_-based structures is still very limited, because according to the best of our knowledge such hetero-structures have still been used for a limited number of practical applications. We expect that the aforementioned titanium oxide TiO_2-x_/TiO_2_-based hetero-structures can find more advanced applications in sensing devices, catalysts, electrode materials, energy storage devices, etc. However, better understanding of the structures formed by the procedure proposed here and their sensing ability, which will meet the requirements of other specific applications, is still required. In addition, more comprehensive understanding of the transition from the conducting metallic titanium-based layer towards the semiconducting TiO_2-x_/TiO_2_-based structure is still crucial in order to achieve desirable semiconducting properties, which are optimal for the applications mentioned above. Therefore, these challenges will be on the agenda of our further studies related to the development of TiO_2-x_/TiO_2_-structure-based materials and the application of these structures for practical purposes.
